# Predictors and mediators of outcome in cognitive behavioral therapy for chronic pain: the contributions of psychological flexibility

**DOI:** 10.1007/s10865-020-00168-9

**Published:** 2020-07-08

**Authors:** Sophia Åkerblom, Sean Perrin, Marcelo Rivano Fischer, Lance M. McCracken

**Affiliations:** 1 Department of Pain Rehabilitation, Skåne University Hospital, Lund, Sweden; 2 Department of Psychology, Lund University, Lund, Sweden; 3 Department of Health Sciences, Lund University, Lund, Sweden; 4 Department of Psychology, Uppsala University, Uppsala, Sweden

**Keywords:** Psychological flexibility, Cognitive behavioral therapy, Chronic pain, Mediator, Predictor

## Abstract

There is now a consensus in the literature that future improvements in outcomes obtained from cognitive behavioral therapy (CBT) for chronic pain will require research to identify patient and treatment variables that help explain outcomes. The first aim of this study was to assess whether pre-treatment scores on measures of psychological (in)flexibility, acceptance, committed action, cognitive (de)fusion, and values-based action predict outcomes in a multidisciplinary, multicomponent, group-based CBT program for adults with chronic pain. The second aim was to assess whether change scores on these same measures mediate outcomes in the treatment program. Participants were 232 people attending treatment for chronic pain. Of the psychological flexibility measures, only pre-treatment scores on the psychological inflexibility scale predicted outcomes; higher scores on this measure were associated with worse outcomes. However, change scores on each of the psychological flexibility measures separately mediated outcomes. The efficacy of CBT for chronic pain may be improved with a greater focus on methods that increase psychological flexibility.

## Introduction

Systematic and meta-analytic reviews find that multicomponent cognitive behavioral therapy (CBT) is superior to treatment as usual and no treatment (but not other active treatments) for adults with chronic pain arising from various conditions (excluding headaches) (Ehde, Dillworth, & Turner, 2014; Williams, Eccleston, & Morley, 2012). Effect sizes for pain and related disability tend to fall in the small to moderate range, and little is known about who is most likely to benefit from this treatment, the appropriate dose, the format (group versus individual), or the necessary treatment components (Turner, Holtzman, & Mancl, 2007; Williams et al., 2012). There is now a general consensus in the literature that improvements in CBT for chronic pain will require research to identify: (a) patient characteristics that predict improvements in key outcomes and (b) therapeutic processes that underlie improvements in these outcomes. It is expected that this information will help to refine and personalize existing treatments (DeRubeis et al., 2014; Gilpin, Keyes, Stahl, Greig, & McCracken, 2017; Kraemer, Wilson, Fairburn, & Agras, 2002; Turner et al., 2007; Williams et al., 2012).

The literature on predictors of outcome in CBT for chronic pain is relatively sparse and the findings quite mixed. Variables that reflect emotional functioning appear most often among identified predictors of treatment outcomes (Gilpin et al., 2017). Attempts to identify demographic predictors have been largely unsuccessful (Gilpin et al., 2017; McCracken & Turk, 2002). Pain-related variables, such as intensity, location, diagnosis, and duration have generated inconsistent findings as predictors (Gilpin et al., 2017; McCracken & Turk, 2002; Turner et al., 2007). Previous efforts to identify active therapeutic processes within CBT for chronic pain have yielded a wide range of candidate variables, such as coping (Jensen, Turner, & Romano, 2001, 2007), self-efficacy (Turner et al., 2007), helplessness (Burns, Johnson, Mahoney, Devine, & Pawl, 1998; Burns, Kubilus, Bruehl, Harden, & Lofland, 2003), partner/family support (Romano, Jensen, Turner, Good, & Hops, 2000; Romano et al., 1995), kinesiophobia (Vlaeyen & Linton, 2000), and pain-related beliefs (Jensen et al., 2001, 2007; Spinhoven et al., 2004; Turner et al., 2007), reflecting the diversity of theoretical models and interventions that characterize CBT (McCracken & Morley, 2014). However, relatively few studies have employed designs or statistical procedures that can identify potential mediators of treatment outcome, a key step in identifying causal mechanisms (Turner et al., 2007).

A coherent theoretically-based set of transdiagnostic and broadly applicable processes that have received attention in the chronic pain literature are those identified in the psychological flexibility model (Hayes, Strosahl, & Wilson, 1999; McCracken & Morley, 2014). Psychological flexibility involves six, core, interactive therapeutic processes: acceptance, cognitive defusion, present focused awareness, self as context, committed action, and values-based action (Hayes et al., 1999). These processes are overlapping in the sense that, theoretically, similar learning history and contexts support subsets of the processes, summarized more broadly as behavior patterns that are open, aware, and engaged (Hayes, Vilatte, Levin, & Hildebrandt, 2011). They are also practically interconnected in the sense that without actively feeling pain (acceptance) one is unlikely to do desired activities that might include pain (values and committed action), and without being able to step back from intimidating experiences (self as context), or see one’s thoughts without being dominated by them (cognitive defusion and present focused awareness), one might not even take the first step. This is born out in evidence that treatments designed to impact on psychological flexibility are associated with significant changes in all of these processes (McCracken & Morley, 2014; Scott, Hann, & McCracken, 2016; Yu, Norton, & McCracken, 2017). At the same time evidence from experimental studies also shows that targeting each individual process yields a significant impact on that process (Levin, Hildebrandt, Lillis, & Hayes, 2012). Furthermore, these separate terms are meaningful because they are clinically useful, each one is associated with a particular set of methods intended to enhance it (Hayes et al., 1999).

There is growing evidence that processes from the psychological flexibility model are related to the severity and impact of chronic pain, particularly in those with relatively high clinical complexity (Baranoff, Hanrahan, Kapur, & Connor, 2013; McCracken & Gutiérrez-Martínez, 2011; Scott et al., 2016; Vowles, Wetherell, & Sorrell, 2009, Vowles, Witkiewitz, Sowden, & Ashworth, 2014; Wicksell, Olsson, & Hayes, 2010). However, with few exceptions, previous studies have focused on the role of one or two processes from the psychological flexibility model, most often acceptance, and in relation to outcomes in treatments specifically focused on increasing psychological flexibility, mainly Acceptance and Commitment Therapy (ACT) (McCracken & Morley, 2014). The historical focus on a limited range of processes arises in part because the development and validation of measures to assess the six processes from the model has lagged behind the implementation of the treatment itself. For example, there were 13 RCTs of ACT for chronic pain published before 2016, but no validated measure of self as context before that same year (Yu et al., 2016).

While traditional CBT approaches for chronic pain do not draw upon the psychological flexibility model, they are widely implemented, and at least one study has found that outcomes in a CBT treatment are mediated by changes in pain acceptance (Åkerblom, Perrin, Rivano Fischer, & McCracken, 2015). Thus, further studies investigating the extent to which separate processes from the psychological flexibility model predict and mediate outcomes in both ACT and traditional CBT for pain are needed (Gilpin et al., 2017). One of two studies to investigate the role of multiple processes from the psychological flexibility model in the treatment of chronic pain was carried out by Vowles et al. (2014). The authors administered self-report measures of acceptance, mindfulness and self-compassion, engagement in valued activities, and psychological flexibility coping to 117 adults receiving ACT at a specialist pain clinic. Using structural equation modelling, the authors found that changes in scores on all of the process measures separately mediated outcomes defined by disability, pain-related anxiety, depression, medical visits, and medication usage. In multiple mediation models, only changes in pain acceptance and mindfulness/self-compassion significantly mediated psychosocial disability, pain-related anxiety, and depression; with acceptance, values engagement, and mindfulness/self-compassion significantly mediating the number of medical visits and medications used (Vowles et al., 2014). In the second study, Scott et al. (2016) administered self-report measures of acceptance, cognitive fusion, de-centering (mindfulness), and committed action to 214 adults undergoing ACT-based treatment for chronic pain at a specialist pain clinic. In multiple regression analyses, change scores on the process measures accounted for 6–27% of the variance in pain outcomes at post-treatment, with acceptance being a significant predictor of all outcomes and de-centering unrelated to outcomes. This pattern was largely maintained at the 9-month follow-up (Scott et al., 2016).

In an earlier cohort of patients drawn from the same specialist pain clinic as the current study, we administered self-report measures of acceptance, life-control, affective distress, and social support at pre-, post-treatment and a 12-month follow-up to 409 adults enrolled in a traditional, multidisciplinary, multicomponent CBT program for chronic pain (Åkerblom et al., 2015). Multilevel modelling revealed that changes in these measures, both individually and together, significantly mediated outcomes (pain intensity, pain interference, and depression). However, the one measure assessing a process from the psychological flexibility model (pain acceptance) was the strongest mediator across the different outcomes (Åkerblom et al., 2015).

The present study builds upon previous findings, and addresses gaps in the literature regarding variables that may influence outcomes in chronic pain treatments. It does this by investigating both predictors and mediators of outcome at long-term follow-up (12 months) using a more comprehensive set of variables from the psychological flexibility model than those previously included, and focusing once again on the context of traditional CBT. First, we assess whether pre-treatment scores on measures of psychological (in)flexibility, acceptance, committed action, cognitive (de)fusion, and values-based action predict outcomes as measured by pain intensity, pain interference, and depression in a multidisciplinary, multicomponent, group-based CBT program for adults with chronic pain. Second, we assess whether change scores from pre-treatment to 12-month follow up on these same measures mediate outcomes in the treatment program. As the processes from the psychological flexibility have both overlapping and distinctive roles in the psychological flexibility model and can be individually targeted in treatment (Hayes et al., 2011; Scott et al., 2016), we investigate the contribution of each process in both univariate and multivariate mediation analyses.

## Methods

### Participants and procedure

Participants were 232 consecutive referrals admitted for treatment to the Pain Rehabilitation Unit at Skåne University Hospital between February 2014 and December 2015. The unit is a government supported, regional, specialist center providing services for adults with chronic pain and related disability. Participants were assessed at baseline (pre-treatment), post-treatment, and again 12 months after discharge. All participants gave informed consent and the study was approved by the Regional Ethical Review Board in Lund, Sweden (2013/381).

### Treatment program

The treatment is an outpatient, CBT program, provided by multidisciplinary teams with training in CBT and broad knowledge of pain rehabilitation. Most of the interventions are group-based and focused on helping the patients develop more adaptive ways of thinking and behaving in relation to pain. The interventions are intended to improve practical skills and knowledge regarding physical exercises, body awareness, and relaxation (physiotherapist); pain and medication (physician); work-related and national insurance issues (social worker); ergonomics, time-use adaptations, problem-solving strategies, and everyday occupational performance (occupational therapist); and thoughts, emotions, behaviors, communication, and goal-setting methods (clinical psychologist). The dominant psychological interventions are psychoeducation, cognitive restructuring, and behavioral activation. Generally, the treatment interventions are guided by a CBT framework and not a psychological flexibility approach. The program is 5 weeks in length with 18 active treatment days (5–7 h per day) and the rest of the days used for home practice. This is followed by a 2-month “homework” phase during which patients work on individual goals with support from the treatment team. At the end of this phase, patients receive two additional days of treatment focusing on progress, difficulties, and future goals.

### Measures

#### Chronic Pain Acceptance Questionnaire-8 (CPAQ-8)

Acceptance was measured with the 8-item CPAQ-8 (Fish, McGuire, Hogan, Stewart, & Morrison, 2010). Each item is rated on a 7-point scale (0 =* never true;* 6=* always true)* and summed to produce a total score. Higher scores reflect greater acceptance of pain. Both the English-language original and the Swedish version (α = 0.80) used in this study have been found to have acceptable reliability and validity (Fish et al., 2010; Rovner, Arestedt, Gerdle, Börsbo, & McCracken, 2014).

#### Chronic Pain Values Inventory (CPVI)

Values-based action was assessed with the 12-item CPVI (McCracken & Yang, 2006). Using the same 6-point scale (0 = *not at all;* 5 = *extremely)*, respondents first rate the level of importance of six separate life domains (family, intimate/close interpersonal relations, friends, work, health, and personal growth/learning) and then rate the level of success they experience in behaving according to their valued life domains. For the purposes of this study only the values success subscale was used. Mean success ratings were obtained for the six life domains, with higher scores representing greater success in living according to one’s values. Similar to the English original, the Swedish version has acceptable reliability (α = .84 for the success subscale) and validity (Åkerblom, Perrin, Rivano Fischer, & McCracken, 2017; McCracken & Yang, 2006).

#### Committed Action Questionnaire (CAQ)

Committed action was assessed with the 18-item CAQ (McCracken, 2013). Each item is rated on a 7-point scale (0 = *never true*, 6 = *always true*), with higher scores reflecting higher levels of goal-oriented, flexible persistence, or committed action. Both the original English version and the Swedish version (α = .89) used in this study have good psychometric properties (Åkerblom, Perrin, Rivano Fischer, & McCracken, 2016; McCracken, 2013).

#### The Psychological Inflexibility in Pain Scale (PIPS)

The PIPS is a measure of the overarching construct of psychological (in)flexibility that is comprised of 8 items assessing pain avoidance and 4 items assessing cognitive fusion (Wicksell, Lekander, Sorjonen, & Olsson, 2010). Each item is rated on a 7-point scale (1=* never true;* 7=* always true)*, yielding a total score and subscale scores for cognitive fusion and pain avoidance. Higher scores indicate greater psychological inflexibility, cognitive fusion, and pain avoidance (respectively). The Swedish language original (used in this study) has been shown to have satisfactory reliability (α = .87) and validity (Wicksell, Lekander, et al., 2010). As pain-related avoidance is also assessed by the CPAQ-8, only the total and cognitive fusion scores were used in this study.

#### Hospital Anxiety and Depression Scale (HADS)

Depression was assessed with the 14-item HADS (Zigmond & Snaith, 1983). Each item (7 anxiety and 7 depression items) is rated from 0 to 3 with separate anxiety and depression scores calculated. Higher scores represent higher levels of depression and anxiety over the past week. The cut-off points for the depression and anxiety subscales are: 0–7 for non-cases, 8–10 for doubtful cases, and 11–21 for clinical cases (Zigmond & Snaith, 1983). Only the depression scale is reported in this study. The English original and Swedish version (α = .82 for the depression subscale) used in this study have acceptable reliability and validity (Lisspers, Nygren, & Soderman, 1997; Zigmond & Snaith, 1983).

#### Multidimensional Pain Inventory Version 2 (MPI)

Pain interference was measured using the 11-item pain interference subscale from the MPI (Version 2) (Rudy, Turk, Zaki, & Curtin,^1989^). The respondent uses a 7-point scale (0 = *never*; 6 = *very often)* to rate the degree to which pain interferes in family and marital functioning, work and work-related activities, and social and leisure activities. A mean interference score is calculated with higher scores indicating greater functional impairment from pain. The MPI has satisfactory levels of reliability (α = .70 to .90) and validity (Kerns, Rudy, & Turk, 1985) and the Swedish version used in this study has shown sensitivity when investigating pain outcomes and satisfactory relationships with other measures of pain-related functioning (Åkerblom et al., 2015).

#### Numerical Rating Scale (NRS)

Pain intensity was assessed with the single-item NRS. Patients are asked to rate their pain over the past week on an 11-point scale (0 = *no pain*; 10 = *worst possible pain*). The NRS has been extensively used in chronic pain settings and is considered a valid measure of pain intensity that is sensitive to the effects of pain treatments (Ferreira-Valente, Pais-Ribeiro, & Jensen, 2011; Jensen & Karoly, 1992).

### Statistical analyses

Descriptive statistics for the proposed predictors, mediators, and outcome measures were produced. Within-subjects effect sizes (pre-to-post-treatment and pre-to-follow-up) were calculated for the mediator and outcome variables using the formula described by Dunlap, Cortina, Vaslow, and Burke (1996). Effect sizes (*Cohen’s d*) were interpreted as small (.2), medium (.5), and large (.8) (Cohen, 1988). Using a conservative approach our power analysis indicated that the sample size of 232 was sufficient to detect a pre-treatment to 12-month follow-up effect size of *d* = .2, with 80% power, and *p* = .05 (Williams et al., 2012).

Predictors are variables that influence outcomes regardless of the treatment under study while “true” moderators are variables that interact with the treatment type to influence outcomes in a particular treatment as compared to control groups (Gilpin et al., 2017; Kraemer et al., 2002). As the present design involved a single treatment group, we only analyzed potential predictors. Specifically, we focused on processes from the psychological flexibility model and contrasted these variables to demographic (age) and baseline pain characteristics (number of pain locations and pain duration), an approach used in previous studies of outcome predictors in patients treated for chronic pain (Gilpin et al., 2017; McCracken & Turk, 2002; Turner et al., 2007).

As total scores on the PIPS are often referred in the literature as an index of the broader construct of psychological (in)flexibility, we assessed whether total scores on the PIPS predicted treatment outcomes in separate regression analyses from those where we evaluated whether different sub-processes from the psychological flexibility model predicted outcomes. In the first set of multiple linear regressions, we explored whether pre-treatment values for age, number of pain locations, pain duration, and psychological inflexibility (independent variables) predicted treatment outcomes as indexed (separately) by the 12-month follow-up scores on pain intensity, pain interference, and depression (dependent variables). In the second set of multiple linear regressions, we included age, number of pain locations, pain duration, acceptance, committed action, cognitive fusion, and values-based action (independent variables) with the same three outcome variables. For both sets of regressions, the baseline value on the outcome measure under investigation was included together with the other independent variables to control for pre-treatment variation in the outcome variables. Sex was not included as a comparator predictor in any of the analyses because the sample was only 14% men (see Table 1 below). Pain diagnosis was also not included since the sample entailed 47 different diagnostic codes often with few participants in each group.

**Table 1:** Participant characteristics, means and within-subjects effect sizes for outcome and mediator variables

	Pre-treatment M (SD)	Post-treatment M (SD)	12-month follow-up M (SD)	Pre-post Cohen’s *d*	Pre-Fup Cohen’s *d*
% Male	14.22				
Age in years	41.60 (9.88)				
% Born in Sweden	76.72				
Pain duration in months	97.72 (94.35)				
# of pain locations Mdn (range)	16.00 (36.00)				
*Outcome*					
Pain intensity	7.26 (1.38)	6.58 (1.79)	6.48 (1.62)	.42	.48
Pain interference	4.71 (.79)	4.17 (.99)	4.06 (1.21)	.58	.55
Depression	9.88 (4.25)	7.11 (4.13)	7.88 (4.64)	.66	.44
*Mediator*					
Psychological inflexibility	60.33 (12.14)	50.77 (12.51)	48.97 (14.13)	.78	.84
Acceptance	17.07 (8.34)	23.91 (8.33)	23.96 (9.77)	−.84	−.71
Committed action	58.95 (14.59)	62.79 (13.31)	62.53 (16.13)	−.27	−.18
Cognitive fusion	22.38 (3.99)	19.84 (4.70)	18.69 (5.04)	.58	.80
Values-based action	1.69 (.92)	2.20 (0.98)	2.25 (1.10)	−.57	−.55

Pain intensity was assessed with the Numerical Rating Scale, pain interference with the Multidimensional Pain Inventory, depression with the Hospital Anxiety and Depression Scale, psychological inflexibility and cognitive fusion with the Psychological Inflexibility in Pain Scale, acceptance with the Chronic Pain Acceptance Questionnaire-8, committed action with the Committed Action Questionnaire, and values-based action with the Chronic Pain Values Inventory

Following recommendations in the literature, we tested a model of mediation based on theory and developed before conducting the analyses, investigated several mediators simultaneously, employed a longitudinal design to address concerns about temporality, used measures with satisfactory psychometric properties, and in a large sample of treated patients (Kazdin, 2007; Maric, Wiers, & Prins, 2012; Thoemmes, 2015). Mediation analyses investigate the effect of the independent variable (X) on the dependent variable (Y) through a possible mediator (M). Studies assessing mediation using data from repeated measurements of the same individuals, and without reference to controls, have not received much attention in the methodology literature although such designs are common. The statistical program used here utilizes a path-analytical framework based on an ordinary least-squares approach to fit models of mediation—both univariate and multivariate to observed data (Montoya & Hayes, 2017). It yields similar results to structural equation models (SEM), but only involve observed (and not latent) variables (Hayes et al., 2017). As applied to the current study, X represents the effect of time in treatment (pre-treatment to 12-month follow-up), with change scores on the mediators (M) and outcome variables (Y) assumed to be influenced by the treatment program. The evidence produced from such approaches is not as definitive as that from studies with control groups, random assignment, and more frequent measurement of both mediators and outcomes, but can still deepen our understanding of these processes of change (Maric et al., 2012). More specifically, we investigated whether pre-treatment to 12-month follow-up changes in scores on the measures of pain intensity, pain interference, and depression, all used as a proxy for the effects of treatment, were mediated by pre-treatment to 12-month follow-up changes in psychological inflexibility, acceptance, committed action, cognitive fusion, and values-based action. We chose to analyze both the mediators and outcome variables from pre-treatment to 12-month follow-up. This approach allowed us to investigate changes during the entire time participants were in contact with the treatment program. Also, there were only minor differences between the post-treatment and 12-month follow-up scores on the outcome and mediator measures under study.

The significance of the indirect effect was tested using the bootstrapping method with bias-corrected estimates (Preacher & Hayes, 2004). The significance of the indirect or mediating effect is directly assessed by the cross-product a*b and confidence intervals are derived from the obtained distribution of a*b scores. The indirect effect is significant at the level specified in the analysis if lower and upper bounds do not contain zero. The algorithm and syntax for SPSS is available online (Montoya & Hayes, 2017). In the first step all mediators were investigated individually. In the next step the sub-processes from the psychological flexibility model (acceptance, committed action, cognitive fusion, and values-based action) were investigated together to test the unique variance accounted for by each mediator in these parallel processes. As with the predictor analyses, we did not include change scores on the measure of psychological inflexibility (PIPS) in the same mediation analyses where we evaluated the sub-processes from the psychological flexibility model. All mediation analyses were based on 5000 bootstrapped samples. All analyses were conducted with SPSS (Version 23).

## Results

### Attrition analyses

Recommended principles were followed when handling incomplete data (Dziura, Post, Zhao, Fu, & Peduzzi, 2013; Little et al., 2012). All participants provided data at pre-treatment and only seven participants dropped out from treatment. In total, 6% of participants failed to provide data at post-treatment and 28% at the 12-month follow-up. As Little’s MCAR test suggested the data were missing completely at random, missing values were imputed at the item level using the Expectation–Maximization method (EM), while all available data were used if data were missing at the variable level (Little, 1988; Schafer & Graham, 2002). Data screening through visual inspection of histograms, normal Q–Q and box plots ensured that items were approximately normally distributed. Using the outlier labelling rule, with 2.2 as a multiplier, outliers (n = 3) were identified, winsorized, and included in all succeeding analyses (Hawkins, 1980; Hoaglin & Iglewicz, 1987).

### Descriptive analyses

Demographic and clinical characteristics are presented in Table 1. The sample consisted of 232 participants. The most prevalent pain diagnoses were fibromyalgia (40.52%), neck-related pain (19.40%), and low back pain (4.74%). Half of the participants (49.54%) suffered from pain complaints for more than 5 years. Based on scores from the HADS depression subscale, 44.59% of the participants screened positive for depression. Means, standard deviations, and within-subjects effect sizes (Cohen’s *d*) for the outcome and mediator variables for all time points, using all available data, are presented in Table 1. Effect sizes were in the small to large range.

### Predictor analyses

Two significant findings emerged when investigating relations between pre-treatment values, as potential predictors, and 12-month follow-up outcomes, adjusting for baseline values of the outcome variables. The results of the first set of multiple linear regression analyses, in which pre-treatment values for age, number of pain locations, pain duration, and psychological inflexibility were regressed on outcomes at 12 months, found that participants who reported higher pain inflexibility at baseline reported significantly worse pain interference (β = .200, t = 2.377, *p* = .019) and depression (β = .158, t = 2.001, *p* = .047) at follow-up. Baseline values on these variables were unrelated to pain intensity scores at follow-up. In the second set of multiple linear regression analyses, using age, number of pain locations, pain duration, acceptance, committed action, cognitive fusion, and values-based action as independent variables with the same three outcome variables, no predictors reached statistical significance.

### Mediation analyses

The results of the univariate mediation analyses are presented in Table 2. Psychological inflexibility, acceptance, committed action, and values-based action were all identified as significant mediators for all outcome variables examined individually. Cognitive fusion was a significant mediator of pain interference and depression only. Taken together, changes on the total score between pre-treatment and the 12-month follow-up on the measure of psychological inflexibility showed the strongest mediating effect for the outcome measures.

**Table 2:** Univariate mediation analyses

Dependent variable	N	Mediator	Results for indirect effects a*b	
			Point-estimate (SE)	95% CI LL, UL
Pain intensity				
	149	Psychological inflexibility	.332 (.127)	.097, .594
	158	Acceptance	.212 (.105)	.013, .425
	149	Committed action	.051 (.033)	.006, .145
	149	Cognitive fusion	.174 (.101)	−.015, .383
	137	Values-based action	.314 (.097)	.155, .536
Pain interference				
	152	Psychological inflexibility	.452 (.074)	.319, .605
	161	Acceptance	.415 (.078)	.275, .579
	152	Committed action	.077 (.044)	.007, .173
	152	Cognitive fusion	.191 (.065)	.072, .326
	140	Values-based action	.239 (.060)	.140, .377
Depression				
	151	Psychological inflexibility	1.573 (.347)	.951, 2.332
	160	Acceptance	1.619 (.326)	1.050, 2.344
	151	Committed action	.332 (.180)	.050, .766
	151	Cognitive fusion	.529 (.274)	.002, 1.105
	138	Values-based action	1.014 (.252)	.606, 1.582

*LL* lower limit, *UL* upper limit. The indirect effect is statistically significant if the confidence interval (CI) does not include zero

Table 3 presents the results of the multivariate analyses assessing the mediating roles of acceptance, committed action, cognitive fusion, and values-based action (and excluding psychological inflexibility) upon pain intensity, pain interference, and depression. When assessing mediators in this multivariate way, the only significant mediator of pain intensity was change scores on the measure of values-based action. Acceptance, committed action, and values-based action together were significant mediators for pain interference and depression, with the most influential mediators being acceptance and values-based action.

**Table 3:** Multivariate mediation analyses including the sub-processes from the psychological flexibility model

Dependent variable	N	Mediator	Results for indirect effects a*b	
			Point-estimate (SE)	95% CI LL, UL
Pain intensity	135			
		Acceptance	.028 (.152)	−.279, .324
		Committed action	.023 (.040)	−.047, .119
		Cognitive fusion	.038 (.121)	−.209, .263
		Values-based action	.283 (.108)	.108, .530
Pain interference	138			
		Acceptance	.262 (.074)	.132, .421
		Committed action	.046 (.025)	.009, .109
		Cognitive fusion	.048 (.074)	−.107, .181
		Values-based action	.103 (.047)	.027, .212
Depression	136			
		Acceptance	.757 (.300)	.233, 1.403
		Committed action	.309 (.139)	.098, .662
		Cognitive fusion	−.195 (.287)	−.833, .280
		Values-based action	.493 (.206)	.134, .958

*LL* lower limit, *UL* upper limit. The indirect effect is statistically significant if the confidence interval (CI) does not include zero

## Discussion

There is a general consensus in the literature that greater efforts are needed to identify active therapeutic processes in CBT approaches for chronic pain (Turner et al., 2007; Williams et al., 2012). The primary aims of this study were to evaluate whether processes from the psychological flexibility model predicted and/or mediated outcomes in a multidisciplinary, multicomponent, group-based CBT program for chronic pain. Overall, significant improvements in pain-related functioning were observed for the treatment program, with uncontrolled effect sizes ranging from small to medium for the outcome variables. These findings correspond with earlier studies from this clinic (Åkerblom et al., 2015) and with the treatment outcome literature more broadly, finding that such programs are empirically-supported and moderately effective forms of psychosocial treatment for chronic pain (Williams et al., 2012).

In relation to whether processes from the psychological flexibility model predicted treatment outcomes, higher baseline (total) scores on the measure of psychological *in*flexibility (comprised of items assessing pain avoidance and cognitive fusion) predicted higher levels of pain interference and depression 1 year after treatment. However, baseline scores on the sub-processes from the psychological flexibility model assessed in this study (acceptance, committed action, cognitive fusion, and values-based action), did not predict any treatment outcomes. The present results can be compared with those of a recent study by Gilpin, Stahl, and McCracken (2018) involving 609 patients treated with ACT for chronic pain (Gilpin et al., 2018). The authors evaluated the predictive role of baseline scores on measures of psychological flexibility (broadly), as well as core processes from the model as indexed by measures of acceptance, committed action, cognitive fusion, and a related concept termed ‘de-centering’. The broad measure of psychological flexibility and the measure of de-centering predicted treatment outcomes, but none of the other core processes from the psychological flexibility model were significant predictors.

The results of the present study and those of Gilpin et al. (2018) suggest that self-report measures of the broader construct of psychological flexibility may work better as predictors of treatment outcome than do measures of individual processes from the psychological flexibility model (Gilpin et al., 2018). Such a conclusion is not necessarily inconsistent with the psychological flexibility model as applied to pain, wherein the six core therapeutic processes are thought to overlap and interact with each other. An alternative interpretation is that further efforts are needed to develop more sensitive methods for detection, or more powerful methods of analysis, of the six core processes from the psychological flexibility model that can help identify whether or not individuals will respond favorably to treatment. At present, brief self-report measures of the broader psychological flexibility construct like the 12-item PIPS (used here) and the 7-item Acceptance and Action Questionnaire-II used in Gilpin et al. (2018) may be useful in identifying patients who may benefit from supplemental interventions that target psychological flexibility more directly (Bond et al., 2011; Gilpin et al., 2018).

The second aim of this study was to investigate whether the same processes from the psychological flexibility model mediated treatment outcomes. When investigating the potential mediators on a univariate level, psychological inflexibility, acceptance, committed action, cognitive fusion, and values-based action all demonstrated mediating effects on outcomes, with scores on the psychological inflexibility scale being the strongest mediator. When investigating the combined, core sub-processes from the psychological flexibility model simultaneously, acceptance, committed action, and values-based action were shown to be significant mediators of treatment outcome. Acceptance and values-based action were the most influential mediators across the different outcomes. These findings add to a small body of literature that have found several core sub-processes from the psychological flexibility model to simultaneously mediate pain outcomes in psychological flexibility-based (ACT) treatments for chronic pain (McCracken & Gutiérrez-Martínez, 2011; Scott et al., 2016; Vowles et al., 2014).

While not a primary aim of the present study, outcomes as indexed by pain intensity, pain interference, and depression 12 months after treatment were not predicted by age, pain duration, or the number of pain locations. These findings add to a larger literature which has failed to find consistent evidence that such variables predict outcomes in traditional CBT programs for chronic pain, at least as examined in group data (Gilpin et al., 2017; McCracken & Turk, 2002; Turner et al., 2007).

Studies of multicomponent CBT programs for chronic pain have not yet revealed which individual treatment components are necessary, and for whom, to achieve improved outcomes (Vowles et al., 2009; Williams et al., 2012). The results from this study and a previous study from the same clinic suggest that there are interventions within this multicomponent program that have beneficial effects on acceptance, committed action, cognitive (de)fusion, values-based action, and psychological (in)flexibility, and in turn on pain-related outcomes (Åkerblom et al., 2015). What is not known, and requires further study, is which of the individual components of standard CBT or psychological flexibility/ACT-based approaches for chronic pain that are linked to these proposed mediators. Studies employing fewer interventions and/or using N-of-1 designs may help identify individual treatment components that best act to increase psychological flexibility. These studies can also focus specifically on patients identified by lower baseline scores on measures of psychological flexibility to identify interventions that are best suited for addressing these processes and improving treatment outcomes.

To date, the focus on potentially important variables to target in CBT has been broad and diverse, possibly too diverse (McCracken & Morley, 2014). Recently, efforts have been made to move beyond disorder specific protocols and division between therapeutic labels and bring different traditions of CBT and evidence-based therapy more generally together within a so-called “process-based” approach, focusing on building interventions around key therapeutic processes of change (Hayes & Hofmann, 2017, 2018). Perhaps, such simplification and integration can be helped by including the psychological flexibility model. Taken together, the results from this study support the notion that the processes from the psychological flexibility model are transdiagnostic and trans-situational. These processes appear to operate in traditional and contextual forms of CBT as well as for a population with diverse problems including both somatic and emotional complaints.

### Limitations

The findings from this study must be viewed within the context of certain limitations. First, we use the term “mediation” recognizing that the strongest evidence for mediation come from treatment studies with random assignment and control groups, and with assessment of both mediator and outcomes at every session to evaluate whether changes in the mediator occur before changes in the outcome variable (temporality). Still, mediation studies fall on a continuum of evidence where studies lacking more than one treatment group and temporality can help identify potential mediators for further investigation (Maric et al., 2012). Second, the findings from this study are based solely on self-report measures. Third, the present sample may be more homogenous than those described in other treatment studies, as it included more women than men and patients who were mostly born in Sweden. Fourth, the PIPS was used to capture psychological (in)flexibility and cognitive (de)fusion. This measure has demonstrated acceptable validity and reliability (Wicksell, Lekander, et al., 2010). However, concerns regarding the internal consistency of the cognitive fusion subscale and the representativeness of the total score as a measure of all aspects of psychological (in)flexibility have been raised in cross-validation studies after this data collection was finished (Barke, Riecke, Rief, & Glombiewski, 2015; Trompetter et al., 2014). Finally, our predictor and mediator findings are based upon a multicomponent treatment delivered by multidisciplinary teams in a specialist center for chronic pain and may not generalize to other clinical settings or treatment approaches.

### Conclusions

A traditional, multidisciplinary, multicomponent CBT program for chronic pain yielded mostly medium effect sizes for pain-related outcomes and potential processes of change. The present results suggest that individuals who had the highest levels of psychological inflexibility before treatment benefitted least well from this approach. In addition, improvements in primary outcomes during this treatment were related to simultaneous increases in psychological flexibility. The efficacy of CBT for patients with chronic pain may be improved with a greater focus on methods that increase psychological flexibility, particularly for patients low on this variable at the start of treatment.
